# Intravenous iron-induced hypophosphatemia and kidney stone disease

**DOI:** 10.1016/j.bonr.2024.101759

**Published:** 2024-03-29

**Authors:** Marlene Panzer, Eva Meindl, Benedikt Schaefer, Sonja Wagner, Bernhard Glodny, Gert Mayer, Andreas Pircher, Christoph Schwarz, Felix Beckmann, Clivia Hejny, Bastian Joachim-Mrosko, Juergen Konzett, Herbert Tilg, Isabel Heidegger, Myles Wolf, Ralf Weiskirchen, Heinz Zoller

**Affiliations:** a Christian Doppler Laboratory for Iron and Phosphate Biology, Austria; b Department of Internal Medicine I, Austria; c Department of Radiology, Austria; d Department of Medicine IV, Austria; e Department of Internal Medicine V, Anichstrasse 35, 6020 Innsbruck, Austria; f Department of Medicine 1, Pyhrn-Eisenwurzen Klinikum Steyr, Sierninger Str. 170, 4400 Steyr, Austria; g Institute of Materials Physics, Helmholtz-Zentrum Hereon, Max-Planck-Str. 1, 21502 Geesthacht, Germany; h Institute of Mineralogy and Petrography, Faculty of Geo- and Atmospheric Sciences, University of Innsbruck, Innrain 52, 6020 Innsbruck, Austria; i Department of Urology, Medical University of Innsbruck, Anichstrasse 35, 6020 Innsbruck, Austria; j Division of Nephrology, Department of Medicine, Duke University School of Medicine and Duke Clinical Research Institute, 40 Duke Medicine Cir Durham, NC 27710-4000, United States of America; k Institute of Molecular Pathobiochemistry, Experimental Gene Therapy and Clinical Chemistry (IFMPEGKC), RWTH University Hospital, Pauwelsstr. 30, 52074 Aachen, Germany

**Keywords:** Urolithiasis, Kidney stone, Hypophosphatemia, IV iron, Hyperphosphaturia, Calcitriol

## Abstract

Patients with Crohn's disease are at increased risk for symptomatic nephrolithiasis. Stones in these patients are most commonly composed of calcium oxalate monohydrate or mixed calcium-oxalate and calcium-phosphate. Precipitation of both minerals depends on urinary pH, calcium, phosphate and oxalate excretion. The present manuscript reports on two patients with Crohn's disease and bowel resection, in whom the onset of symptomatic urolithiasis occurred after repeated infusions of ferric carboxymaltose – a drug, which is known to cause hyperphosphaturia. The present study shows that ferric carboxymaltose-induced hyperphosphaturia can be associated with kidney stone formation and symptomatic urolithiasis, especially in patients treated with calcitriol. Calcitriol has been shown to mitigate ferric carboxymaltose-induced secondary hyperparathyroidism and hyperphosphaturia, but is known to increase urinary calcium excretion. Chemical analysis of recovered stones revealed that they were mixed calcium oxalate and phosphate stones. Ring-like deposition of iron detected by spatially resolved elemental analysis using laser ablation-inductively coupled plasma mass spectrometry, showed that the stones also contained iron. Based on our findings, we propose that patients with inflammatory bowel disease requiring intravenous iron therapy should be carefully monitored for the development of hypophosphatemia and urolithiasis. If hypophosphatemia occurs in such patients, calcitriol should be used with caution.

## Highlights

• Treatment of iron deficiency with certain IV irons could lead to nephrolithiasis.
• IV‑iron induced hypophosphatemia treated with phosphate increases kidney stone risk.
• Calcitriol treatment accelerates stone-formation.
• Phosphate traces may be missed in mixed calcium-oxalate and phosphate stones by XRD.
• Hyperoxaluria in patients with bowel disease may be an additional risk factor.

## Graphical abstract

**Figure:**
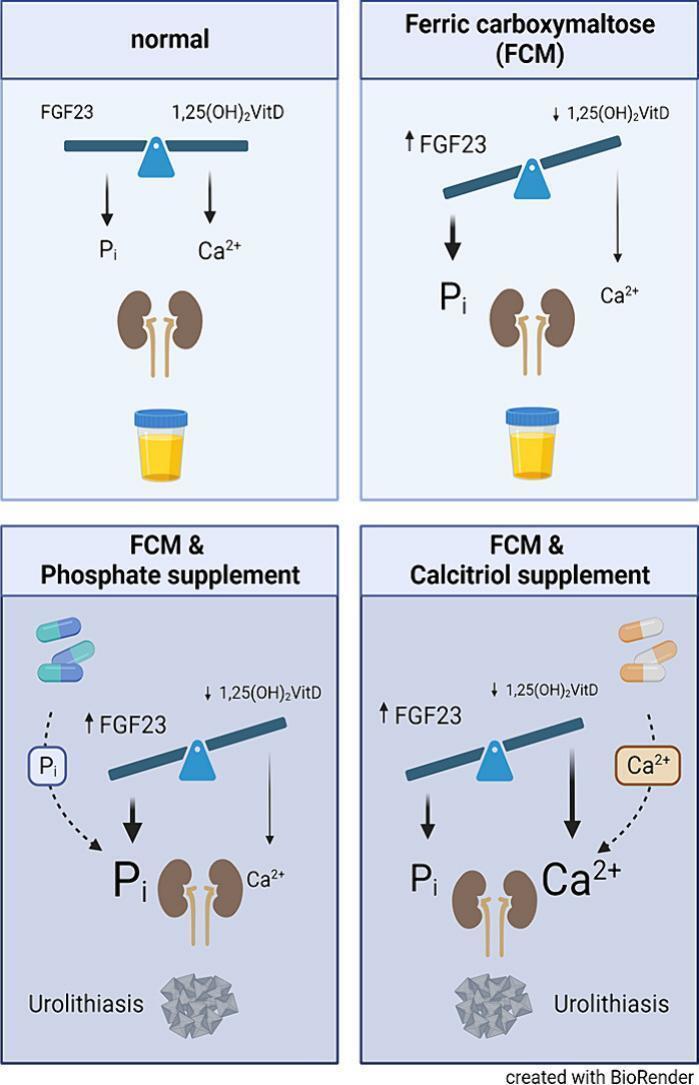

## 1 Introduction

The incidence of kidney stones ranges from 114 to 720 per 100,000 person-years and is determined by geographical, climatic, dietary, and genetic factors (Thongprayoon et al., 2020). Investigation of stone composition is the basis for management. The majority of symptomatic kidney stones are composed of calcium oxalate and/or calcium phosphate. Accordingly, hyperoxaluria and high urinary phosphate excretion are risk factors for the development of kidney stones (Walker et al., 2013). Recent studies have shown that calcium phosphate accelerates nucleation of calcium oxalate crystal aggregation and stone formation (Xie et al., 2015; Walker, 2019).

Both kidney stones and osteomalacia are typical manifestations of the genetic renal phosphate wasting condition hypophosphatemic rickets with hypercalciuria (HHRH - caused by mutations in *SLC34A3*), where high urinary phosphate excretion is associated with hypercalciuria (Taguchi et al., 2017). In contrast, patients with X-linked hypophosphatemia (XLH) or tumor-induced osteomalacia (TIO), who also have high urinary phosphate excretion but low urinary calcium, rarely present with urolithiasis (Bosman et al., 2022). This phenotypic difference in urinary calcium excretion is caused by differences in circulating 1,25-(OH)_2_ vitamin D, which is regulated by fibroblast growth factor-23 (FGF23). FGF23 controls plasma phosphate by regulating renal phosphate excretion and inhibiting the activation of 25OH-vitamin D to 1,25-(OH)_2_-vitamin D. FGF23 is low in patients with HHRH and in patients with *SLC34A1* defects, which are both associated with high levels of 1,25-(OH)_2_-vitamin D, resulting in high urinary calcium excretion (Patzer et al., 1999; Letavernier et al., 2018). In contrast, syndromes with high FGF23 (e.g. XLH and TIO) are associated with reduced 1,25-OH-vitamin D. Such patients with high FGF23 are therefore protected from urinary stone formation because low 1,25-OH-vitamin D (calcitriol) reduces urinary calcium excretion (Meyer and Pike, 2020) (Table S1).

High FGF23, hypophosphatemia, hyperphosphaturia, which are hallmarks of XLH and TIO, can also be caused by certain intravenous iron formulations – in particular ferric carboxymaltose (FCM), iron polymaltose or rarely high doses of saccharated iron oxide (Schaefer et al., 2020). High FGF23 in IV iron-induced hypophosphatemia is also associated with low 1,25-(OH)_2_-Vitamin D and secondary hyperparathyroidism (Wolf et al., 2020; Daude et al., 2021). Therefore, high doses of calcitriol have been used to treat IV iron-induced hypophosphatemia (Vasquez-Rios et al., 2021; Anand and Schmid, 2017). Here we report a series of patients in whom symptomatic kidney stone disease was triggered by FCM-induced hypophosphatemia and secondary hyperparathyroidism. The study was approved by the Ethics Committee at the Medical University of Innsbruck (protocol 1285/2022, 17th of Novermber 2022) and written informed consent was obtained.

## 2 Case series

**Fig. 1:**
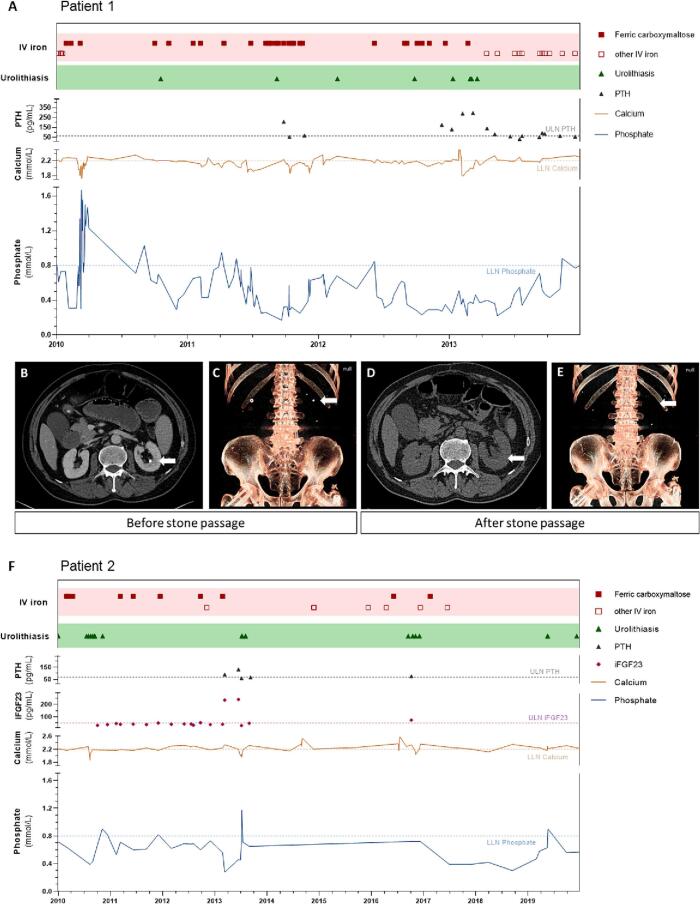
Clinical and treatment course of Patient 1 **(A)** and Patient 2 **(F)**. Correlation between ferric carboxymaltose infusions (*red squares*), serum phosphate concentrations, serum calcium concentration, PTH, iFGF23 and symptomatic kidney stone events/colics (*green triangles*). Lower limit of normal (LLN) for serum calcium and serum phosphate (*blue horizontal line*) and upper limit of normal (ULN) of PTH and intact FGF23 are marked. **(B-E)** Serial computed tomography scans (patient 1). Axial CT scan **(B, D)** showing a calcification at the tip of the papilla in the left kidney in Jan 2013, which disappeared after stone passage indicating that stone formation was initiated in collecting duct plugs and not in Randall plaques. 3D reconstructions of CT scans shown in **(C, E)** confirming disappearance of renal calculi.

Patient 1 was a man with Crohn's disease who underwent ileocecal resection at the age of 24 years. Due to chronic disease activity, he was treated with methylprednisolone and developed recurrent ileal stenosis at the age of 35 years requiring several surgical resections. The patient subsequently reported recurrent episodes of symptomatic urolithiasis (Fig. 1A). Of note, renal colic occurred during a period of repeated intravenous infusions of ferric carboxymaltose, causing severe hypophosphatemia (lowest value 0.17 mmol/L). Recurrent treatment by intravenous phosphate infusions (20 mmol each) as well as oral supplementation of 38.4 mmol of phosphate in three divided doses per day did not sustainably correct hypophosphatemia, but likely further increased urinary phosphate excretion. After repeated infusions of FCM, intact FGF23 was elevated to 187 pg/mL (upper limit of normal 60 pg/mL). Hypophosphatemia gradually improved after FCM was switched to ferric derisomaltose and no further episodes of symptomatic urolithiasis occurred for the subsequent 4 years.

**Fig. 2:**
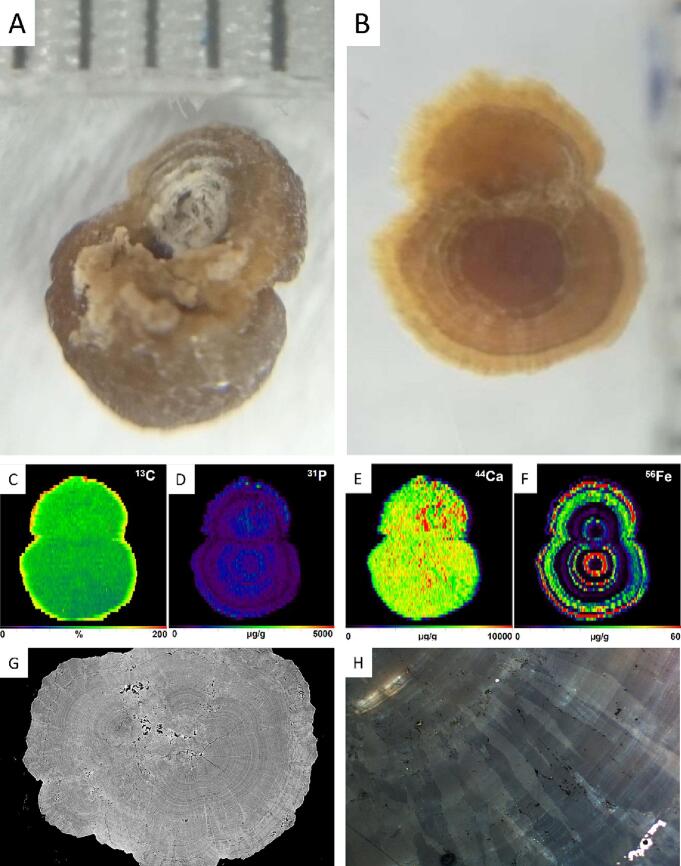
**Stone analysis. (A)** Epiluminenscence microscopic image of the stone (distance between vertical lines is 1 mm) **(B)** Epiluminenscence microscopic image of a cut and polished section of the epoxy resin embedded stone showing the ring like stone structure. **(C**—F**)** Quantitative analysis of ^13^C, ^31^P, ^44^Ca and ^56^Fe of the stone surface by laser ablation-inductively coupled plasma mass spectrometry. **(G)** SRμCT analysis showing aggregate structure of the stone with multiple intersected rings. The CT investigation was performed using Synchrotron Radiation at the beamline P05 operated by Hereon at the Synchrotron Radiation facility PETRA III at DESY, Hamburg, Germany. **(H)** Stone section under polarized light showing radial and concentric stone structure.

X-ray diffraction (XRD)-analysis of the patient's kidney stones suggests that they consist of almost pure Ca-oxalate monohydrate. Synchrotron radiation-based micro-computer tomography (SRμCT) revealed a complex growth pattern of the stones with several nucleation sites that started to grow independently to form spherical aggregates (Fig. 2G). During continuing growth, some of these aggregates eventually amalgamated. Optical microscopy using polarized light shows that the growth patterns within these spherical aggregates are characterized by a radial arrangement of spindle-like crystals up to several hundred μm in length in varying orientation (Fig. 2B). Polarized light microscopy further shows that crystal growth occurs layer-upon-layer with a thickness of individual layers typically <10 μm to form a tree ring-like pattern (Fig. 2H). Laser ablation-inductively coupled plasma mass spectrometry (LA-ICP-MS) mapping of a stone showed a concentric oscillatory compositional zoning in phosphorous and iron (Fig. 2D and F)) and a patchier zoning in calcium (Fig. 2E). Oscillatory zoning is particularly well developed for iron, which is attributed to an increased availability of iron in urine during treatment of the patient with ferric carboxymaltose. Iron was detectable at concentrations of up to 35 mg/L in the first urine void after the intravenous infusion of 1 g of FCM in this patient. This ring-like pattern was only found in a single patient when the same analysis was repeated in a series of 13 consecutive stone patients without prior exposure to FCM.

Diagnostic CT scans showed calcifications at the tip of the renal papillae, which were not associated with plaques on ureteroscopy (Fig. 1B and C). Disappearance of the calcifications on subsequent imaging (Fig. 1D and E) further suggests that kidney stone formation was initiated in the collecting ducts as Randall plaques were excluded (compare Fig. 1C and E).

Patient 2 was a 63-year-old man with Crohn's disease since age 22 who presented with symptomatic urolithiasis due to two kidney stones measuring 8 and 13 mm, which required treatment by extracorporeal shock-wave lithotripsy (ESWL). The patient had a history of several ileal resections, which resulted in recurrent bleeding from the ileo-colic anastomosis requiring repeated intravenous iron infusions. Four months after iron therapy had been changed from iron sucrose to ferric carboxymaltose and four years after his presentation and ESWL, the patient again developed renal colic requiring surgery.

At this time, the patient presented with hypophosphatemia of 0.49 mmol/L (Fig. 1F). Treatment of hypophosphatemia with oral and IV phosphate could not sustainably correct hypophosphatemia. After secondary hyperparathyroidism (PTH 138 ng/L – reference range 15–65 ng/L) was diagnosed, treatment with 0.25 μg calcitriol was started. FCM was discontinued after it had been identified as the cause of the patient's high intact FGF23 of 233 pg/mL (upper limit of normal 60 pg/mL). Despite discontinuation, the patient did not recover from hypophosphatemia and presented with recurrent renal colic and passage of kidney stones. Calcitriol was therefore discontinued, which resulted in a reduction in the frequency of symptomatic urolithiasis.

Analysis of the patient's kidney stone by X-ray diffraction showed that the stone was mainly composed of calcium oxalate monohydrate. Elemental analysis confirmed that the main constituent was calcium, while the stone also contained 2.9 mg/g phosphate and trace amounts of iron (Table S2).

## 3 Discussion

Inflammatory bowel diseases (IBD), especially Crohn's disease, and short bowel syndrome are risk factors for hyperoxaluria and urolithiasis. The present case series shows that in patients who underwent bowel resection, treatment of iron deficiency with FCM and treatment of FCM-associated hypophosphatemia with calcitriol or phosphate could represent additional risk factors for symptomatic kidney stone disease in predisposed patients (Daude et al., 2021).

In the patients reported here several risk-factors coincided that are known to accelerate the mineralization in the urinary tract. Formation of calcium-containing stones is a complex process that is favoured by the excretion of highly concentrated minerals in urine (Alelign and Petros, 2018). The fact that cessation of calcitriol treatment reduced the frequency of symptomatic stone disease, suggests that high urinary calcium excretion triggered by calcitriol treatment was a major contributing factor. Another important risk factor present in both patients is the fact that both patients had undergone bowel resections, which can cause hyperoxaluria (Witting et al., 2021). Iron could also have contributed to stone formation in both patients, because iron concentration could be high in urine directly after IV infusion - especially when labile iron complexes are used. Other factors such as local hypoxia at the tip of the papilla, or hyperoxaluria after bowel resection cannot be modified. In contrast, hyperphosphaturia is a modifiable risk factor, which can be avoided by using stable IV iron formulations of low hypophosphatemic potential.

Recent studies confirmed that high urinary phosphate excretion after FCM is caused by an increase in circulating FGF23 (Schaefer et al., 2021; Schaefer et al., 2022). Although X-ray diffraction analyses showed that the kidney stones in both patients were mainly composed of calcium oxalate, elemental analysis identified appreciable amounts of phosphorus and traces of iron. Spatially resolved analysis by LA-ICP-MS of embedded kidney stones revealed that iron was present in ring-like patterns. The latter finding suggests that iron was present in urine at high concentrations and could have resulted in limited displacement of calcium in oxalate crystals. The contribution of iron to stone formation is not clear and other IV iron formulations could be found in high concentrations in the urine, which might be a relevant cofactor that warrants further study. Identification of phosphate on elemental analysis, which did not co-localize with iron in LA-ICP-MS analysis, demonstrated that stones are composed of aggregates of calcium oxalate monohydrate and calcium phosphate. This finding suggests that high urinary phosphate excretion could have accelerated calcium oxalate stone formation. It has been shown that calcium phosphate accelerates nucleation of calcium oxalate crystal formation (Xie et al., 2015).

In both patients, symptomatic kidney stone development was temporally associated with ferric carboxymaltose-induced hypophosphatemia and high concentrations of circulating FGF23. The association between high FGF23, hypophosphatemia, hyperphosphaturia, hypocalcitriolemia, hypocalcemia as well as high parathyroid hormone concentrations was recently summarized as 6H-syndrome (Schaefer et al., 2020). Parathyroid hormone (PTH) further increases renal phosphate excretion and can persist for months after discontinuation of FCM (Schaefer et al., 2022). A limitation of the study is that no longitudinal data on urinary calcium excretion are available.

Treatment with oral and IV phosphate did not sustainably correct hypophosphatemia, because FGF23 remained elevated causing renal phosphate wasting. Administration of phosphate thereby further increases urinary phosphate excretion, which could have resulted in accelerated kidney stone formation in Case 1. As hypophosphatemia was associated with hyperparathyroidism in Case 2, this patient was treated with calcitriol. As calcitriol increases urinary calcium excretion, this could have accelerated crystal growth and stone formation in Case 2 (Santos et al., 1986).

A distinctive feature of urolithiasis associated with FCM-induced hypophosphatemia could be calculus formation as collecting duct plugs at the tip of the kidney papillae (Daude et al., 2021). Accordingly, sequential CT imaging showed that after stone passage no papillary calcifications remained. In contrast, patients and animal models of renal calcifications secondary to high urinary phosphate excretion are often associated with nephrocalcinosis, which was not present in our patients.

**Fig. 3:**
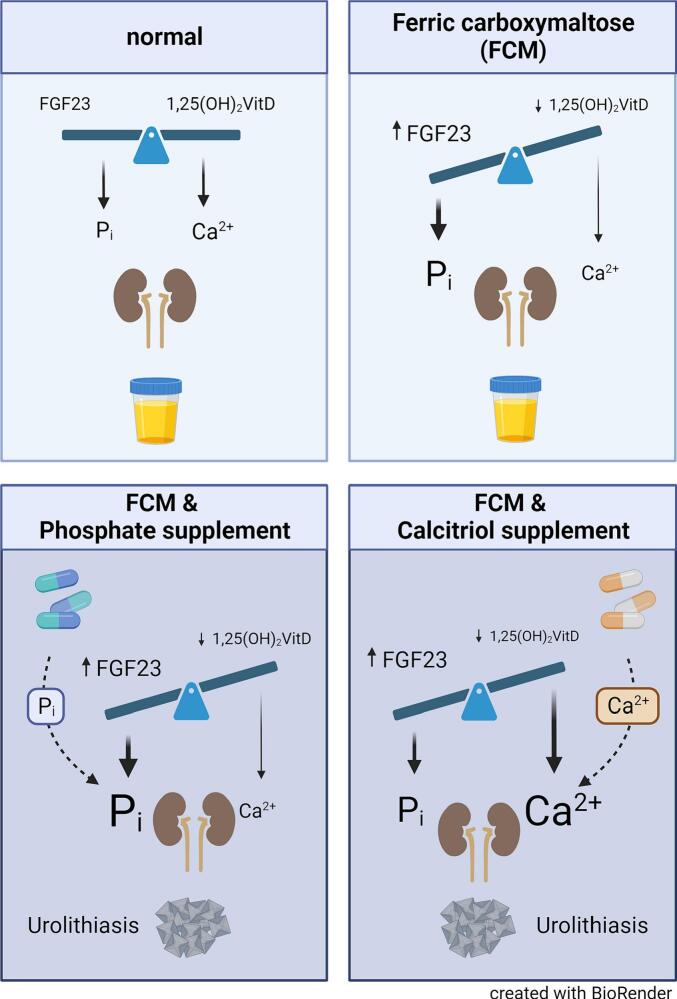
**Upper left panel:** Under normal conditions, adequate FGF23 and calcitriol (1,25(OH)_2_ VitD) cause normal urinary phosphate (P_i_) and calcium (Ca^2+^) excretion without precipitation of either calcium-phosphate or calcium oxalate. **Upper right panel:** Ferric carboxymaltose (FCM) can cause an increase in intact FGF23, resulting in increased urinary phosphate excretion. High FGF23 also inhibits calcitriol production, causing reduced intestinal calcium absorption, mild hypocalcemia and low urinary calcium excretion. **Lower left panel:** Treatment of FCM-induced hypophosphatemia with oral or IV phosphate results in increased urinary phosphate excretion, which can trigger calcium phosphate precipitation and urolithiasis. **Lower right panel:** Treatment of FCM-induced hypocalcemia and secondary hyperparathyroidism with calcitriol supplementation causes increased intestinal absorption of calcium and urinary calcium excretion, which can also trigger urolithiasis.

In conclusion, the present study shows that symptomatic urolithiasis can be triggered by hyperphosphaturia in patients with FCM-induced hypophosphatemia and secondary hyperparathyroidism. Although in such patients secondary hyperparathyroidism can be mitigated by calcitriol, this treatment appears to further increase the risk of kidney stone formation (Fig. 3). Studies have shown that patients without chronic kidney disease (CKD) or mild renal impairment CKDG1–3 may develop hypophosphatemia when treated with FGF23-inducigng IV iron infusions such as FCM, iron polymaltose or saccharated iron oxide. To detect and prevent complications from high FGF23 in such patients serum phosphorous, alkaline phosphatase and bone-specific alkaline phosphatase should be measured. Ideally, IV iron formulations with a low risk of hypophosphatemia should have preference for the treatment of iron deficiency and calcitriol should be used with caution in patients with IV iron-induced hypophosphatemia. Such low-risk IV iron formulations, that can also be used in CKD stage 4/5 patients include ferric derisomaltose, low-molecular weight iron dextran, and ferrumoxytol, which is not available in Europe (Boots and Quax, 2022).

## CRediT authorship contribution statement

**Marlene Panzer:** Writing – original draft, Visualization, Methodology, Formal analysis, Data curation, Conceptualization. **Eva Meindl:** Writing – original draft, Visualization, Methodology, Formal analysis, Data curation, Conceptualization. **Benedikt Schaefer:** Writing – review & editing, Visualization, Funding acquisition, Data curation, Conceptualization. **Sonja Wagner:** Writing – review & editing, Investigation, Formal analysis. **Bernhard Glodny:** Writing – review & editing, Visualization, Validation, Formal analysis. **Gert Mayer:** Writing – review & editing, Validation, Conceptualization. **Andreas Pircher:** Writing – review & editing, Validation, Methodology. **Christoph Schwarz:** Writing – review & editing, Conceptualization. **Felix Beckmann:** Writing – review & editing, Visualization, Methodology, Investigation, Formal analysis, Data curation. **Clivia Hejny:** Writing – review & editing, Methodology, Formal analysis. **Bastian Joachim-Mrosko:** Writing – review & editing, Methodology, Formal analysis, Data curation. **Juergen Konzett:** Writing – review & editing, Visualization, Methodology, Formal analysis, Data curation. **Herbert Tilg:** Writing – review & editing, Validation. **Isabel Heidegger:** Writing – review & editing, Validation, Methodology. **Myles Wolf:** Writing – review & editing, Conceptualization. **Ralf Weiskirchen:** Writing – review & editing, Visualization, Methodology, Data curation, Conceptualization. **Heinz Zoller:** Writing – review & editing, Writing – original draft, Visualization, Supervision, Project administration, Methodology, Funding acquisition, Conceptualization.

## Declaration of competing interest

Marlene Panzer has no conflict of interest to declare in relation to this work.

Eva Meindl has no conflict of interest to declare in relation to this work.

Benedikt Schaefer has received honoraria for lecturing from Vifor, the manufacturer of ferric carboxymaltose.

Sonja Wagner has no conflict of interest to declare in relation to this work.

Bernhard Glodny has received grant support and consulting fees from Vifor, the manufacturer of ferric carboxymaltose.

Gert Mayer has no conflict of interest to declare in relation to this work.

Andreas Pircher has no conflict of interest to declare in relation to this work.

Christoph Schwarz has no conflict of interest to declare in relation to this work.

Felix Beckmann has no conflict of interest to declare in relation to this work.

Clivia Hejny has no conflict of interest to declare in relation to this work.

Bastian Joachim-Mrosko has no conflict of interest to declare in relation to this work.

Juergen Konzett has no conflict of interest to declare in relation to this work.

Herbert Tilg has no conflict of interest to declare in relation to this work.

Isabel Heidegger has no conflict of interest to declare in relation to this work.

Myles Wolf has no conflict of interest to declare in relation to this work.

Ralf Weiskirchen has no conflict of interest to declare in relation to this work.

Heinz Zoller has received grant support and honoraria for lecturing and consulting fees from Vifor, the manufacturer of ferric carboxymaltose.

## Data Availability

Data will be made available on request.

## Appendix A Supplementary data

Supplementary tables Image 1
