# Supplementary material for: Intravenous iron-induced hypophosphatemia and kidney stone disease
Source: Bone Rep. 2024 Mar 29;21:101759. doi: 10.1016/j.bonr.2024.101759 (PMC10999795; doi:10.1016/j.bonr.2024.101759)
Supplement: Supplementary file 1 — Supplementary tables [file mmc1.docx]

# Supplementary Tables

Suppl. Table 1: Effect of FGF23 on disease formation of the kidney.

| **Low FGF23 associated hypophosphatemia**  **(HHRH, genetic defects in *SLC34A3*)** | **High FGF23 conditions**  **(6H Syndrome, ADHR, XLH, ARHR Type 1/2, TIO)** |
| --- | --- |
| Low FGF23 | High FGF23 |
| Up-regulation of *CYP27B1* | Down-regulation of *CYP27B1* |
| High 1,25(OH)_2_ vitamin D | Low 1,25(OH)_2_ vitamin D |
| Increased Ca^2+^ absorption | Reduced Ca^2+^ absorption |
| High iCa | Low iCa |
| High urinary Ca^2+^ excretion | Low urinary Ca^2+^ excretion |
| Urolithiasis | Nephrocalcinosis? |

Suppl. Table 2: Elemental analysis of the stones by inductively coupled plasma mass spectrometry of entire stones, showing that the molar ratio between calcium and phosphorous is ca 1:100 and that the substitution of Ca by Fe is <1:1000.

| Element | Patient 1  (μg/g kidney stone) | Patient 2  (μg/g kidney stone) |
| --- | --- | --- |
| Calcium | 260,000 | 271,000 |
| Phosphorus | 2,590 | 2,930 |
| Sodium | 1,790 | 1,890 |
| Iron | 64 | 358 |
| Magnesium | 350 | 317 |
| Strontium | 90 | 92.7 |
| Zinc | 65 | 29.9 |
